# Supplementary material for: Contrasting geochemical and fungal controls on decomposition of lignin and soil carbon at continental scale
Source: Nat Commun. 2023 Apr 19;14:2227. doi: 10.1038/s41467-023-37862-6 (PMC10115774; doi:10.1038/s41467-023-37862-6)
Supplement: Supplementary file 1 — Supplementary Information [file 41467_2023_37862_MOESM1_ESM.pdf]

Supplementary Information for

Contrasting geochemical and fungal controls on decomposition of lignin and soil carbon at continental scale

Wenjuan Huang[1]†, Wenjuan Yu[1]†*, Bo Yi[1], Erik Raman[2], Jihoon Yang[2], Kenneth E. Hammel[3,4], Vitaliy I. Timokhin[5], Chaoqun Lu[1], Adina Howe[2], Samantha R. Weintraub-Leff[6], Steven J. Hall[1]

[1] Department of Ecology, Evolution, and Organismal Biology, Iowa State University, Ames, Iowa, USA.

[2] Department of Agricultural and Biosystems Engineering, Iowa State University, Ames, Iowa, USA.

[3] U.S. Forest Products Laboratory, Madison, Wisconsin, USA.

[4] Department of Bacteriology, University of Wisconsin, Madison, Wisconsin, USA.

[5] Great Lakes Bioenergy Research Center, University of Wisconsin, Madison, Wisconsin, USA.

[6] National Ecological Observatory Network, Battelle, Boulder, Colorado, USA.

* Corresponding author, Wenjuan Yu, Email address: wjyu@iastate.edu

† These authors contributed equally: Wenjuan Huang, Wenjuan Yu

This supplementary file includes:

Supplementary Methods

Supplementary Notes

Supplementary Tables 1–6

Supplementary Figs. 1–14

Supplementary References

Supplementary Methods

Preparation of litter and lignin mixture. Synthetic guaiacyl (softwood type) lignin with either a ^{13}C label (99 atom%) or natural abundance C at the C_β position of each C_9 substructure were prepared as described in Kirk and Brunow[1]. Lignins were fractionated by gel permeation chromatography to obtain a molecular mass greater than 1000 Da. The synthetic lignins were dissolved in acetone/water (4:1) and added to finely ground litter of *Andropogon gerardi* (big bluestem, a C_4 grass) in a 1:25 mass ratio of lignin to litter. The mixture was evaporated in a fume hood, which allows lignins to precipitate on the litter surface. Each subunit of the synthetic lignin had a molecular mass of 197, so the ^{13}C label represented 13/197 of total lignin mass. The mineralization of C_β to CO_2 is a conservative diagnostic of lignin degradation as scission of the lignin polymer is required for the release of C_β. The lignin concentration in *A. gerardi* is 20% by mass, as measured by solid-state ^{13}C nuclear magnetic resonance (NMR) spectroscopy and a molecular mixing model[2]. The total lignin mass in the litter (naturally occurring lignin + added synthetic lignin) for each soil sample was 23.1%. More details on preparation and characterization are given in Hall et al.[3] and Huang et al.[4].

Partitioning of decomposed C sources in the lab incubation. We used a two-source mixing model to determine the fractional contribution of the added C_4 litter-derived C to total C mineralization (f_{litter}) from each soil sample[4,5].

$$f_{litter} = \frac{x(^{13}\text{C})_{\text{soil+litter}} - x(^{13}\text{C})_{\text{soil}}}{x(^{13}\text{C})_{\text{litter}} - x(^{13}\text{C})_{\text{soil}}} \quad (1)$$

Here, $x(^{13}C)_{\text{soil+litter}}$ is the measured atom fraction $^{13}CO_2$ from the soil + litter + unlabeled lignin treatment; $x(^{13}C)_{\text{soil}}$ is the measured atom fraction $^{13}CO_2$ from the soil alone (control); $x(^{13}C)_{\text{litter}}$ is -10.6‰, accounting for a ~2‰ enrichment of carbohydrates relative to the bulk leaf

tissue (-12.6‰)[6]. This assumption was strongly supported by the evidence from our previous study[2], where the same litter used here was incubated with two soils according to the same protocol used in our current study. The $\delta^{13}C$ of CO_2 produced from decomposing litter C agreed closely with the assumed $\delta^{13}C$ values of carbohydrate, and carbohydrates were shown to be the main source of decomposed C (>70% of C loss), as indicated by ^{13}C NMR data[2]. Because decomposition of the synthetic lignins accounted for < 0.1 % of total decomposed C, the influence of unlabeled synthetic lignin on $\delta^{13}C$ of respired C can be neglected in the soil + litter + unlabeled lignin treatment. Rather, the unlabeled lignin was included to account for any effects of the added synthetic lignin on litter decomposition. The C decomposition from the litter was calculated by multiplying f_{litter} by the mean CO_2 produced from the samples in the soil + litter + unlabeled lignin and soil + litter + $^{13}C_{\beta}$-labeled lignin treatments for each sample.

We used another two-source mixing model to determine the fractional contribution of the $^{13}C_{\beta}$-labeled lignin to total C decomposition (f_{lignin}):

$$f_{lignin} = \frac{x(^{13}\mathrm{C})_{\mathrm{soil+litter+13C\ lignin}} - x(^{13}\mathrm{C})_{\mathrm{soil+litter}}}{x(^{13}\mathrm{C})_{\mathrm{lignin}} - x(^{13}\mathrm{C})_{\mathrm{soil+litter}}} \quad (2)$$

Here, $x(^{13}C)_{soil+litter+13C\ lignin}$ is the measured atom fraction $^{13}CO_2$ of samples from the soil + litter + $^{13}C_{\beta}$-labeled lignin treatment; $x(^{13}C)_{soil+litter}$ is as described above; and $x(^{13}C)_{lignin}$ is 0.99. The C decomposition from the $^{13}C_{\beta}$-labeled lignin was calculated by multiplying f_{lignin} by the mean CO_2 produced from the soil + litter + unlabeled lignin and soil + litter + $^{13}C_{\beta}$-labeled lignin treatments for each sample.

Calculation of lignin C decomposition in the field incubation. After about 1 y of field incubation, we found that the total C concentration in some samples was higher than the initial values, which might be due to the growth of fungal hyphae or other C inputs, such as dissolved

organic C, into the mesh bags during the field incubation. Indeed, visible hyphal growth into the bags was observed in several cases. This violates the assumptions of the simple two-source mixing models described above, so we accounted for these new C inputs as follows. We first determined the mean increase in soil C in samples from each site by calculating the difference between measurements of the initial soil C and soil C remaining in the samples from the unlabeled lignin treatment. Then, we subtracted this value from the mean total C remaining measured in samples from both treatments (i.e., soil+litter+unlabeled lignin and soil+litter+$^{13}C_{\beta}$-lignin) at each site, which we defined as the corrected total C remaining. Using the corrected total C value in subsequent calculations occasionally led to implausibly large values of lignin mass, so we compared these values to total C remaining measured in the individual samples in the labeled lignin treatment. If the mean corrected total C remaining was smaller than the measured total C remaining in the labeled lignin, we used this value in subsequent calculations; otherwise, we used the measured value from the individual sample.

The soil C remaining in the soil + litter + unlabeled lignin samples was determined using a two-source mixing model as described in equation (1), where f_{litter} represents the fraction of litter-derived organic C to total C remaining; $x(^{13}\text{C})_{\text{soil+litter}}$ represents atom fraction ^{13}C in samples from the unlabeled lignin treatment; $x(^{13}\text{C})_{\text{soil}}$ is the mean value of the atom fraction ^{13}C in samples from the four points that were used for the lab incubation; and, $x(^{13}\text{C})_{\text{litter}}$ is 0.010919 measured by isotope ratio mass spectrometry at Iowa State University. The fractional contribution of the $^{13}C_{\beta}$-labeled lignin to total C was determined by equation (2), where f_{lignin} is the fraction of lignin C to total C remaining; $x(^{13}\text{C})_{\text{soil+litter+13C lignin}}$ is the atom fraction ^{13}C in the samples from the labeled lignin treatment; $x(^{13}\text{C})_{\text{soil+litter}}$ is atom fraction ^{13}C in the samples from the unlabeled lignin treatment; and, $x(^{13}\text{C})_{\text{lignin}}$ is the atom fraction ^{13}C in the $^{13}C_{\beta}$-labeled

synthetic lignin. The $x(^{13}\mathrm{C})_{\mathrm{lignin}}$ (equal to 11.2) was calculated empirically based on C isotope mixing models as described below, and is equivalent to the above-mentioned $x(^{13}\mathrm{C})_{\mathrm{lignin}}$ of 0.99 for the $^{13}\mathrm{C}_{\beta}$ position of the lignin subunit when the whole lignin unit has the molar mass of 197 and C concentration of 59.6%.

$$f_{\mathrm{lignin\_in\_litter}} = \frac{x(^{13}\mathrm{C})_{\mathrm{litter+13C\ lignin}} - x(^{13}\mathrm{C})_{\mathrm{litter}}}{x(^{13}\mathrm{C})_{\mathrm{lignin}} - x(^{13}\mathrm{C})_{\mathrm{litter}}} \quad (3)$$

Here, $f_{\mathrm{lignin\_in\_litter}}$ (equal to 0.0377) is the fraction of synthetic lignin C to total C in the mixture of litter and lignin (25:1) with litter C of 39.9% and 39.1% C in the $^{13}\mathrm{C}_{\beta}$-lignin. The $x(^{13}\mathrm{C})_{\mathrm{litter}}$ and $x(^{13}\mathrm{C})_{\mathrm{litter+13Clignin}}$ measured 0.010916 and 0.0147237, respectively, by an elemental analyzer (Elementar Analysensysteme GmbH, Hanau, Germany) and continuous flow isotope ratio mass spectrometer (Sercon Ltd., Cheshire, UK) at the UC Davis Stable Isotope Facility. The lignin C remaining after the 1-y field incubation was calculated by multiplying f_{lignin} by total C concentration in the samples of soil+litter+$^{13}\mathrm{C}_{\beta}$-labeled lignin or the corrected value after accounting for new C input as described above. The C decomposition from soil, litter and lignin was expressed as a percentage of the initial C mass in each pool. Total SOC decomposition in one SRER soil sample after the 18-month incubation was assumed to be 99%, as measured decomposition exceeded initial SOC, likely due to heterogeneity between subsamples with relatively low C concentrations (~4 mg C g^{-1}) used in the lab incubation and for SOC measurement.

Supplementary Statistical Methods. We used random forest models (RFMs) to explore possible nonlinear relationships among predictors and C decomposition variables[7]. Variables were not standardized for an easier interpretation of the RFM partial dependence (PD) plot, which showed the marginal effect of each predictor on the predicted response variable. Lab

lignin decomposition was log10 transformed and the same predictors as in the initial LMMs were included in the initial RFMs. Unimportant predictors were removed from models with Z-score < 5 in the "Boruta" package[8]. Some predictors were further removed from final models based on root mean square error (RMSE) and R^2 using repeated (N = 100) cross validation. The RFMs were not overfit as indicated by an overfitting ratio > 10 in the "rfUtilities" package[9]. RFM was applied with 1,000 trees, with other options sticking to default parameters in the "randomForest" package[10]. The RFM performance was evaluated by R^2, and model overfitting was examined using the "rfUtilities" package. Variable importance was assessed using increase of mean squared error (%IncMSE) when a given variable is randomly permuted; a larger increase in MSE illustrates greater importance of the permuted variable.

To further explore variable interactions in the RFM, we first measured the overall interaction strength (H-statistic) for each variable in the model[11]. The H-statistic is 0 if there is no interaction at all, and an H-statistic of 1 between two predictors means that each single PD function is constant and the effect on the prediction only comes through the interaction. Next, as we were particularly interested in how decomposition drivers might change across the large climate gradient, we calculated two-way interactions among MAP or MAT and other predictors and presented two-way PD plots with H-statistic > 0.05.

We further used generalized additive mixed models (GAMMs) to test whether the relationships among predictors and C decomposition variables were linear, using the "mgcv" package[12]. GAMMs are a variant of LMMs that allow for non-linear relationships and fit a statistically optimum degree of curvature to the data. The degree is quantified using estimated degrees of freedom, where a value of 1 is a linear relationship and the higher the value, the more nonlinear the relationship is. We fit the GAMMs using the same predictors as in the LMMs, also

accounting for the random effect of site and using a smooth function based on thin plate regression splines and an identity link function (default values in the mgcv package).

We also investigated whether putative lignin-degrading fungal organisms, i.e., white-, brown-, and soft-rot fungi identified in the FUNGuild database[13], were linked to lignin decomposition. Similarly, we used PC1 or PC2 of a principal coordinate analysis based on Hellinger distances among samples from the lab incubation at 14 months calculated using only the ASV abundances of the identified "rot" fungi to represent their overall composition. We summed relative abundance of the identified "rot" fungi for each sample. Pearson correlations were then performed among lab lignin decomposition and overall composition and summed abundance of the "rot" fungi. We also used Pearson correlations to examine relationships among lab lignin decomposition and individual fungal genera occurring in more than 10 samples.

Supplementary Notes

Pairwise relationships of C decomposition with biogeochemical predictors. Cumulative decomposition of lignin and litter at the end of lab incubation tended to have similar pairwise relationships with biogeochemical predictors, whereas lab lignin and SOC decomposition had generally opposite pairwise relationships with those same biogeochemical predictors (Supplementary Fig. 10). We assessed these relationships after 6, 12 and 18 months of cumulative decomposition (Supplementary Figs. 10 and 11), and they changed little over time, such that we focused our subsequent analysis on the 18-month (571 d) dataset. Different axes of fungal community composition correlated with lignin and litter vs. SOC decomposition. MAP was significantly and positively related to lignin decomposition but negatively related to SOC decomposition ($P < 0.01$). Lignin decomposition had a significantly negative relationship with

NO_3^--N after 1 m of incubation ($P < 0.01$), while litter decomposition was positively related to total N, NH_4^+-N, NO_3^--N and inorganic N (NH_4^+-N + $NO3^-$-N) after 18 months ($P < 0.05$ for NH_4^+-N and $P < 0.01$ for the other variables). Soil pH was significantly and negatively related with lignin decomposition but positively related with SOC decomposition ($P < 0.01$). The silt+clay content and some soil metals ($Fe_{cd\text{-}ox}$ and Mn_{cd}) had significant and positive relationships with lignin decomposition but negative relationships with SOC decomposition ($P < 0.01$). Fungal quantity had a positive relationship with lignin and litter decomposition but a negative relationship with SOC decomposition. Ten fungal genera occurring in more than 10 samples had significant ($P < 0.05$) correlations with lignin decomposition (Supplementary Table 4): positive for *Pleotrichocladium, Geomyces, Trichocladium, Mycena, Hypochnicium, Solicoccozyma,* and *Mortierella* and negative for *Cladophialophora, Pseudofabraea,* and *Glarea*. Both the composition of known “rot” fungi and their summed relative abundance was insignificantly ($P > 0.05$, data not shown) correlated with lignin decomposition.

Compared with lab lignin decomposition, field lignin decomposition was also closely related to some predictors within climatic, N, geochemical and microbial categories, although not all relationships were consistent. Specifically, fungal quantity had a positive relationship with field lignin decomposition, consistent with the lab lignin decomposition. However, the field lignin decomposition had a negative relationship with MAP and a positive relationship with soil pH, which was inconsistent with the lab lignin decomposition. Field lignin and lab lignin decomposition had opposite pairwise relationships with several biogeochemical predictors, such that field lignin and lab SOC decomposition also had several biogeochemical predictors in common (Supplementary Fig. 10). However, most of these relationships disappeared in the statistical models after other predictors were accounted for (Fig. 4). The discrepancy may also

have been due to wider ranges of predictor and response variables in the field samples (such as fungal abundance and Fe_{ox}), and the strikingly different climate conditions in the field than in the lab (Supplementary Fig. 1 and Supplementary Table 1).

Importance of biogeochemical predictors for C decomposition in the RFMs. Compared with the LMM, a slightly higher proportion of variation in lab lignin decomposition ($R^2 = 0.51$) was explained in the RFM. The optimal RFM included the same predictors as the LMM (except for Fe_{ox}), with the addition of bacterial quantity, MAP, and Fe_{cd-ox}. Predicted lignin decomposition decreased with pH and increased with most other predictors (Supplementary Fig. 6). However, predicted lignin decomposition varied nonlinearly with soil C/N, with a minimum at C/N = 20, and it also varied nonlinearly with Fe_{cd-ox}, with a minimum at approximately 7 mg Fe g^{-1}.

The RFM of field lignin decomposition included the same nine predictors shown in the LMM, and explained 39% of the variation in field lignin decomposition. As observed in the LMM, predicted field lignin decomposition in the RFM generally increased with Mn_{cd} and Ca_{cd} (except for a decrease between 2.5–6 mg g^{-1} Ca_{cd}) and decreased with increasing Fe_{ox} and Al_{ox} (Supplementary Fig. 7). Unlike lab lignin decomposition, predicted field lignin decomposition showed more complex relationships with MAT, with a null relationship below 15°C and a positive relationship at higher temperatures. Also, predicted field lignin decomposition decreased with increasing MAP, and Fe_{cd-ox} was not an important predictor. Similar to the lab lignin decomposition, predicted field lignin decomposition varied nonlinearly with soil C/N, but with a minimum at a slightly lower C/N value of 13, as opposed to 20 (Supplementary Fig. 7).

The optimal RFM of lab litter decomposition explained 60% of the variation, and included all of the 17 biogeochemical predictors except for silt+clay and soil C/N. The predictors

shared with the LMM all had directionally similar correlations with litter decomposition, illustrated by comparing model coefficients (Fig. 4) and the RFM partial dependence plot (Supplementary Fig. 8). Fe_{ox}, Al_{ox}, and pH had negative relationships with litter decomposition in the RFM while most other predictors had positive relationships (Supplementary Fig. 8).

The optimal RFM of SOC decomposition explained 55% of the variation and included soil C/N and all geochemical and climatic predictors. The relationships of these predictors with SOC decomposition was similar with those in the LMM. Predicted SOC decomposition generally decreased with soil C/N, reactive minerals and metals, and MAP, and increased with MAT and pH in the RFM (Supplementary Fig. 9).

The overall interaction strengths of predictors were all < 0.25 (Supplementary Table 6) in lab decomposition RFMs, indicating relatively weak interactions. Despite generally higher lab lignin decomposition in soils with MAP > 1200 mm, relationships among lab lignin decomposition and predictors were often similar between < 1200 mm MAP and > 1200 mm MAP (Supplementary Fig. 12). Similarly, relationships among lab lignin decomposition and predictors were generally similar between < 3 °C MAT and > 3 °C MAT (Supplementary Fig. 12). For lab litter and SOC decomposition, the influence of other biogeochemical predictors was generally consistent across the climatic gradients (Supplementary Figs. 13 and 14). We did not explore interactions in the field lignin RFM given the absence of some predictors that were included in the lab lignin RFM.

Supplementary Table 1. Soil, ecosystem, and climatic attributes of 20 NEON sites.

Site ID	Plot ID*	Soil order	NLCD class	Latitude (°)	Longitude (°)	MAT (°C)	MAP (mm)	O horizon† (cm)	SOC in 0–15 cm‡ ($mg\ g^{-1}$)	SOC in 15–30 cm ($mg\ g^{-1}$)
BONA	004	Inceptisols	Evergreen Forest	65.19	-147.54	-3	262	12	88	15
CPER	006	Alfisols	Grassland/Herbaceous	40.81	-104.75	8.6	344	0	10	6
DSNY	023	Entisols	Woody Wetlands	28.09	-81.42	22.4	1213	0	35	19
GRSM	001	Inceptisols	Deciduous Forest	35.68	-83.53	11.3	1657	0	51	21
HARV	015	Inceptisols	Deciduous Forest	42.54	-72.18	8	1210	7	34	20
KONZ	001	Mollisols	Grassland/Herbaceous	39.08	-96.56	12.4	869	0	44	–
LENO	006	Inceptisols	Woody Wetlands	31.81	-88.19	18.1	1383	0	20	14
NIWO	041	Inceptisols	Grassland/Herbaceous	40.05	-105.58	0.3	971	1	42	24
ONAQ	004	Aridisols	Shrub/Scrub	40.19	-112.47	8.7	380	0	13	10
OSBS	002	Entisols	Evergreen Forest	29.70	-81.96	20.9	1306	0	9	5
PUUM	017	Entisols	Evergreen Forest	19.57	-155.32	12.7	2657	3	251	175
SCBI	004	Alfisols	Deciduous Forest	38.90	-78.15	11.9	1076	0	48	16
SJER	045	Mollisols	Evergreen Forest	37.12	-119.73	16.8	508	0	15	10
SRER	002	Aridisols	Shrub/Scrub	31.87	-110.86	18.8	399	0	5	4
TALL	004	Ultisols	Evergreen Forest	32.96	-87.43	17.2	1376	0	25	11
TOOL	002	Gelisols	Dwarf Scrub	68.63	-149.35	-9	316	21	48	44
UNDE	006	Spodosols	Deciduous Forest	46.25	-89.51	4.3	799	0	27	17
WOOD	003	Mollisols	Grassland/Herbaceous	47.12	-99.24	4.9	494	0	39	26
WREF	071	Andisols	Evergreen Forest	45.82	-121.96	8.6	2488	2	28	19
YELL	006	Mollisols	Shrub/Scrub	44.97	-110.56	1.3	829	1	41	30

MAT, mean annual temperature; MAP, mean annual precipitation; SOC, soil organic carbon measured from the 4 sampling points for lab incubation. MAT and MAP data are obtained from[14].

*Plot ID refers to the specific "distributed base plot" in the NEON sampling design which was sampled for our study.

†The depth in O horizon represents the mean value of organic horizon depth from the 16 sampling points (note that only the mineral soil horizons were incubated in our study).

‡Depth is with respect to the beginning of the mineral horizon (i.e., the organic horizon is not included)

Supplementary Table 2. Description of biogeochemical predictors used in this study, arranged by conceptual category.

Category	Variable	Unit	Note
C decomposition	Lignin CO_2	%	Lignin C decomposition incubated in the lab relative to initial added lignin C mass
	Litter CO_2	%	Litter C decomposition incubated in the lab relative to initial added litter C mass incubated in the
	Soil CO_2	%	Soil C decomposition incubated in the lab relative to initial soil organic C mass
	Field lignin C decomposition	%	Lignin C decomposition incubated in the field relative to initial added lignin C mass
Geochemical	pH	–	–
	Silt+Clay (%)	%	Summed percentage of silt- and clay-sized particles
	Al_{ox}	mg g^{-1} soil	Short-range-ordered (SRO) phases of Al and/or organo-Al complexes extracted by ammonium oxalate
	Fe_{ox}	mg g^{-1} soil	Short-range-ordered (SRO) phases of Fe and/or organo-Fe complexes extracted by ammonium oxalate
	Fe_{cd-ox}	mg g^{-1} soil	Crystalline phases of Fe (the difference between Fe extracted by citrate/dithionite and ammonium oxalate)
	Fe_{HCl}	mg g^{-1} soil	Dissolved and adsorbed Fe(II) as well as dissolved, organically-complexed Fe(III), and a reactive fraction of Fe(III) minerals extracted by HCl
	Mn_{cd}	mg g^{-1} soil	Short-range-ordered (SRO) phases of Mn and organo-Mn complexes extracted by citrate/dithionite
	Ca_{cd}	mg g^{-1} soil	Exchangeable Ca and Ca in organo-Fe associations extracted by citrate/dithionite
Microbial	Fungal composition	–	Principal components (PC2 and PC1) of principal coordinate analysis based on Hellinger distance of fungal species abundance at 14 month for lignin/litter and soil C decomposition, respectively.
	Fungal Chao1 richness	–	Residual of ASV Chao1 index in relation to the square root of the number of total sequences within each sample at 14 month
	Fungal quantity	gene copies g^{-1} soil	ITS rRNA gene copy number in soil at 0 month
	Bacterial quantity	gene copies g^{-1} soil	16S rRNA gene copy number in soil at 0 month
	Fungal-to-bacterial ratio (F/B)	–	ITS rRNA gene copy number divided by 16S rRNA gene copy number at 0 month
N-related	Total N	mg g^{-1} soil	Soil total N
	C/N	–	Ratio of soil organic carbon (SOC) to total soil N
	Ammonium	μg N g^{-1} soil	Soil ammonium concentrations in soil + litter mixture samples after 1, 9, and 18 months
	Nitrate	μg N g^{-1} soil	Soil nitrate concentrations in soil + litter mixture samples after 1, 9, and 18 months

	Inorganic N	$\mu g\ N\ g^{-1}$ soil	Soil inorganic N concentrations (sum of ammonium and nitrate concentrations) in soil + litter mixture samples after 1, 9, and 18 months
Climatic	MAT	^{o}C	Mean annual temperature
	MAP	mm	Mean annual precipitation

Supplementary Table 3. Estimated degrees of freedom of predictors in the generalized additive mixed models (GAMMs), where a value >1 indicates an increasingly nonlinear relationship.

Predictor	Lab lignin decomposition	Lab litter decomposition	Lab SOC decomposition	Field lignin decomposition
pH	1	1	1	1
Silt+Clay	1	–	1	–
Al_{ox}	–	–	1	1
Fe_{ox}	1	1	–	1
Fe_{cd-ox}	–	–	–	1
Fe_{HCl}	–	1	–	–
Mn_{cd}	1	1	1	1
Ca_{cd}	1	–	1.45	1
Fungal composition	1	1	–	–
Fungal Chao1	–	–	–	–
Fungal quantity	2.99	2.24	–	–
Bacterial quantity	–	2.60	–	–
Fungal-to-bacterial ratio	–	–	–	–
Bulk N	–	3.01	–	1
Bulk C/N	1	–	3.23	1
MAT	1	1	1	1
MAP	–	–	1.69	1

Supplementary Table 4. Fungal genera occurring in more than 10 samples that had significant (*P* < 0.05) correlations with lab lignin decomposition.

Pearson correlation coefficient	Phylum	Class	Order	Family	Genus	No. of samples
0.22	*Ascomycota*	*Dothideomycetes*	*Pleosporales*	*Melanommataceae*	*Pleotrichocladium*	21
-0.16	*Ascomycota*	*Eurotiomycetes*	*Chaetothyriales*	*Herpotrichiellaceae*	*Cladophialophora*	42
-0.18	*Ascomycota*	*Leotiomycetes*	*Helotiales*	*Dermateaceae*	*Pseudofabraea*	105
-0.16	*Ascomycota*	*Leotiomycetes*	*Helotiales*	*Helotiaceae*	*Glarea*	10
0.34	*Ascomycota*	*Leotiomycetes*	*Thelebolales*	*Pseudeurotiaceae*	*Geomyces*	24
0.17	*Ascomycota*	*Sordariomycetes*	*Sordariales*	*Chaetomiaceae*	*Trichocladium*	50
0.26	*Basidiomycota*	*Agaricomycetes*	*Agaricales*	*Tricholomataceae*	*Mycena*	10
0.25	*Basidiomycota*	*Agaricomycetes*	*Polyporales*	*Podoscyphaceae*	*Hypochnicium*	16
0.17	*Basidiomycota*	*Tremellomycetes*	*Filobasidiales*	*Piskurozymaceae*	*Solicoccozyma*	127
0.28	*Mortierellomycota*	*Mortierellomycetes*	*Mortierellales*	*Mortierellaceae*	*Mortierella*	142

Supplementary Table 5. The predictors used in each of the initial models, after pruning for collinearity

Lab lignin decomposition	Lab litter decomposition	Lab SOC decomposition	Field lignin decomposition
pH	pH	pH	pH
Silt+Clay	Silt+Clay	Silt+Clay	—
Al_{ox}	Al_{ox}	Al_{ox}	Al_{ox}
Fe_{ox}	Fe_{ox}	Fe_{ox}	Fe_{ox}
$Fe_{cd\text{-}ox}$	$Fe_{cd\text{-}ox}$	$Fe_{cd\text{-}ox}$	$Fe_{cd\text{-}ox}$
Fe_{HCl}	Fe_{HCl}	Fe_{HCl}	—
Mn_{cd}	Mn_{cd}	Mn_{cd}	Mn_{cd}
Ca_{cd}	Ca_{cd}	Ca_{cd}	Ca_{cd}
Fungal composition	Fungal composition	Fungal composition	—
Fungal Chao1	Fungal Chao1	Fungal Chao1	—
Fungal quantity	Fungal quantity	Fungal quantity	—
Bacterial quantity	Bacterial quantity	Bacterial quantity	—
Fungal-to-bacterial ratio	Fungal-to-bacterial ratio	Fungal-to-bacterial ratio	—
Bulk N	Bulk N	—	Bulk N
Bulk C/N	Bulk C/N	Bulk C/N	Bulk C/N
MAT	MAT	MAT	MAT
MAP	MAP	MAP	MAP

Supplementary Table 6. Overall interaction strength of predictors in the random forest models (RFMs) as represented by Friedman's H-statistic, where 0 indicates no interaction and 1 indicates that an effect is entirely explained by an interaction.

Predictor	Lab lignin decomposition	Lab litter decomposition	Lab SOC decomposition
pH	0.12	0.05	0.07
Silt+Clay	0.14	–	0.14
Al_{ox}	–	0.05	0.11
Fe_{ox}	–	0.05	0.11
$Fe_{cd\text{-}ox}$	0.10	0.06	0.09
Fe_{HCl}	–	0.10	0.10
Mn_{cd}	0.11	0.08	0.11
Ca_{cd}	0.10	0.05	0.11
Fungal composition	0.19	0.10	–
Fungal Chao1	–	0.12	–
Fungal quantity	0.14	0.12	–
Bacterial quantity	0.16	0.17	–
Fungal-to-bacterial ratio	–	0.05	–
Bulk N	–	0.13	–
Bulk C/N	0.11	–	0.25
MAT	0.05	0.04	0.10
MAP	0.12	0.06	0.06

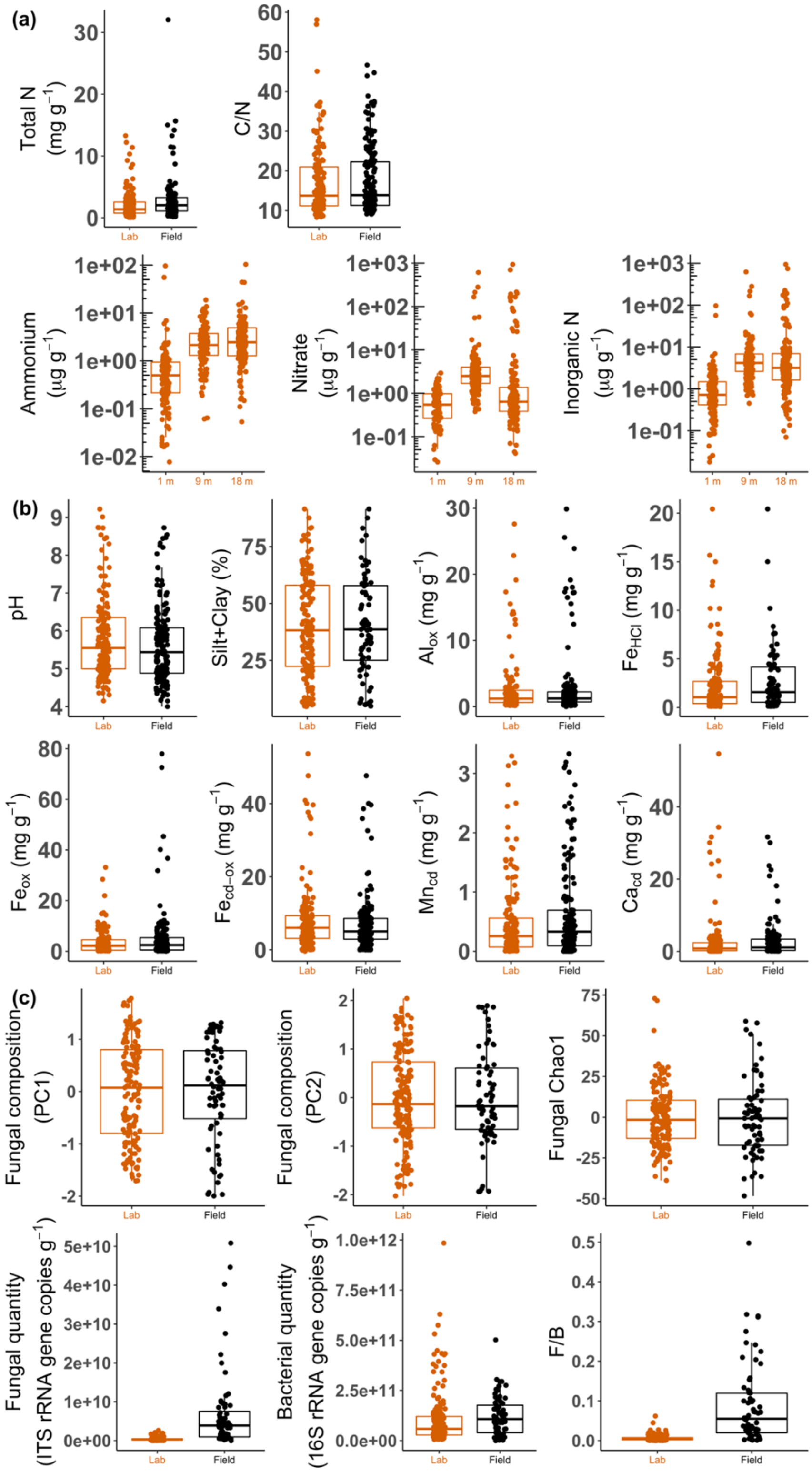

Supplementary Fig. 1 Scatter and box plots of nitrogen (N)-related (a), geochemical (b), microbial (c) predictors for the lab carbon decomposition (orange) and field lignin

decomposition (black) across 20 NEON sites. Predictors correspond to those in Supplementary Table 2. In the boxplots, thick lines represent the median, boxes represent the interquartile range, whiskers represent points within 1.5 times the interquartile range, and dots represent raw data. For the lab incubation data, n = 156 biologically independent samples for N-related and geochemical predictors, and n = 160 biologically independent samples for field incubation data on N-related and geochemical predictors. For data on fungal composition after 9 months of incubation, n = 155, and for approximately 1 year of field incubation, n = 72 biologically independent samples. For microbial quantity (qPCR) after 9 months of lab incubation, n = 156 biologically independent samples, and for approximately 1 year of field incubation, n = 65 biologically independent samples. Source data are provided as a Source Data file.

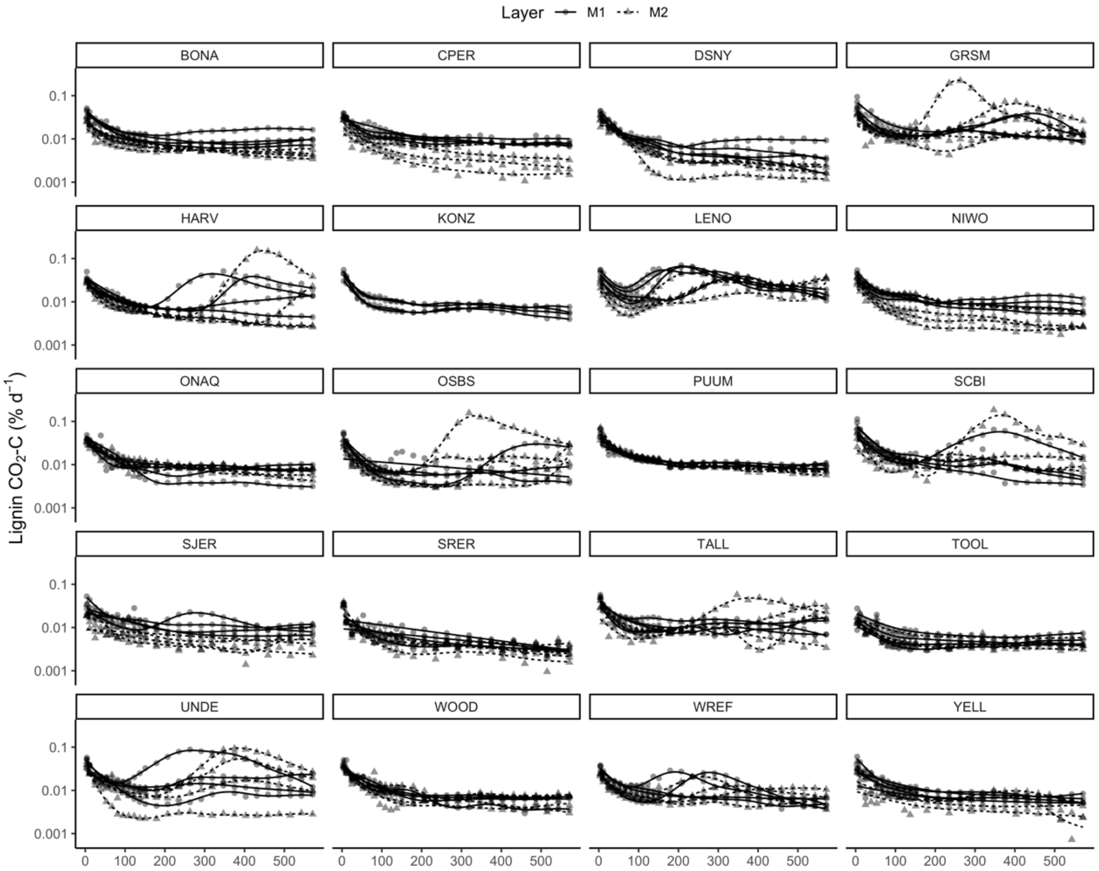

Supplementary Fig. 2 Decomposition (CO_2-C) rate from synthetic lignin carbon (C) in each lab incubated sample at 20 NEON sites. Note the base-10 logarithmic y-axis scale for lignin C decomposition rate. Lines (solid line for M1 layer and dotted line for M2 layer) are fit by generalized additive mixed models (GAMMs). M1 (circle) and M2 (triangle) denote mineral soil samples from 0-15 cm and 15-30 cm depths, respectively. Source data are provided as a Source Data file.

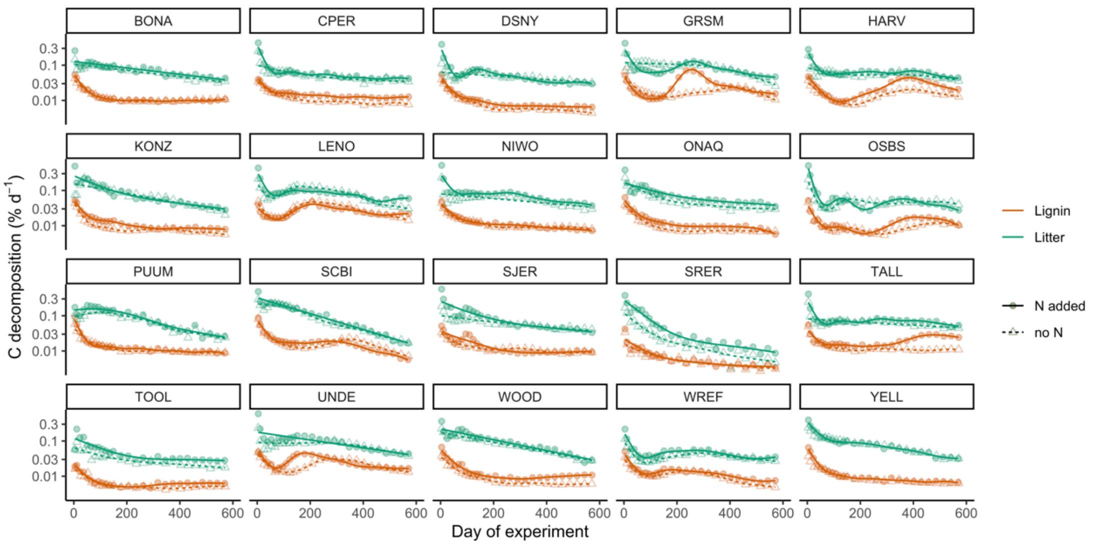

Supplementary Fig. 3 Effects of nitrogen (N) addition on carbon (C) decomposition from synthetic lignin and litter in lab-incubated samples from 20 NEON sites, expressed as a percentage of the initial C mass in each pool. Note that the base-10 logarithmic y-axis scale for C decomposition rate. Lines are fit by generalized additive mixed models (GAMMs). Each point represents the mean decomposition rate from four sampling points at each site (n = 4 biologically independent samples per site). This experiment only included samples from 0-15 cm depth. The circle represents N addition, and the triangle represents the control (no N addition). The lines in orange and green represent lignin and litter, respectively. Source data are provided as a Source Data file.

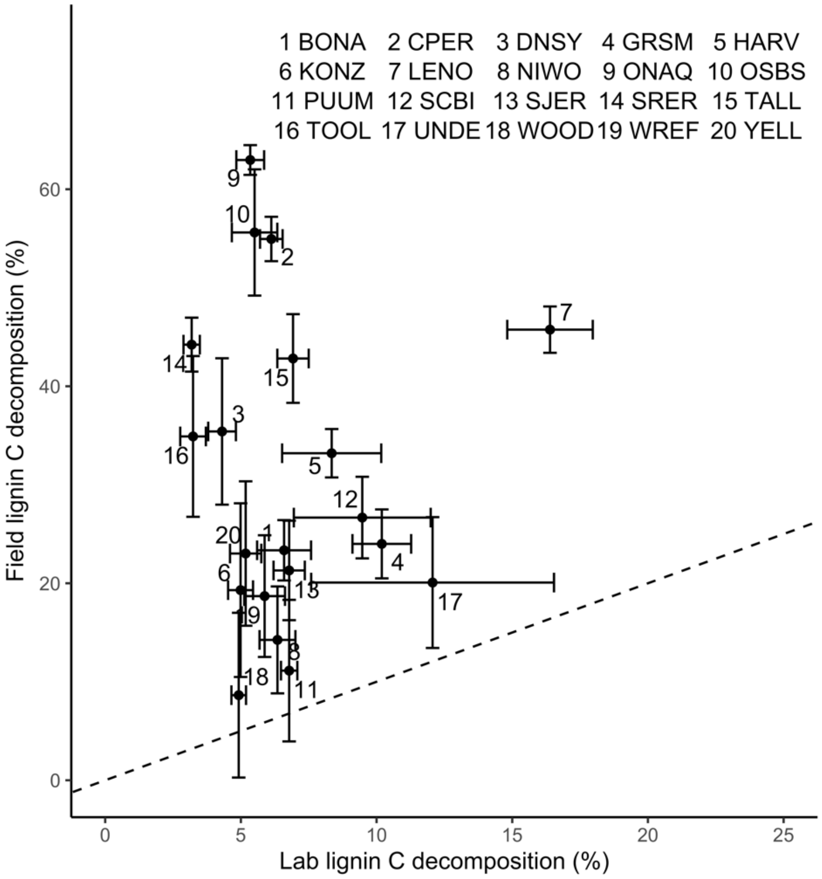

Supplementary Fig. 4 Cumulative lignin C decomposition as a percent of initial mass in samples incubated in the field and lab at 20 NEON sites. The error bars indicate standard errors (SE). Numbers correspond to means from each NEON site according to the legend, denoted by four-letter site IDs (Fig. 1 and Supplementary Table 1). Data are presented as mean values ± SE. Note different x-axis and y-axis scales. The four letters represent the site ID (Fig. 1 and Supplementary Table 1). For lab incubation data, n = 4 biological independent samples per point; for field incubation data, n = 8 biological independent samples, except for some sites (n = 7 for PUUM, n = 6 for SCBI, n = 3 for SJER, n = 7 for WOOD) due to the missing samples in the field. Source data are provided as a Source Data file.

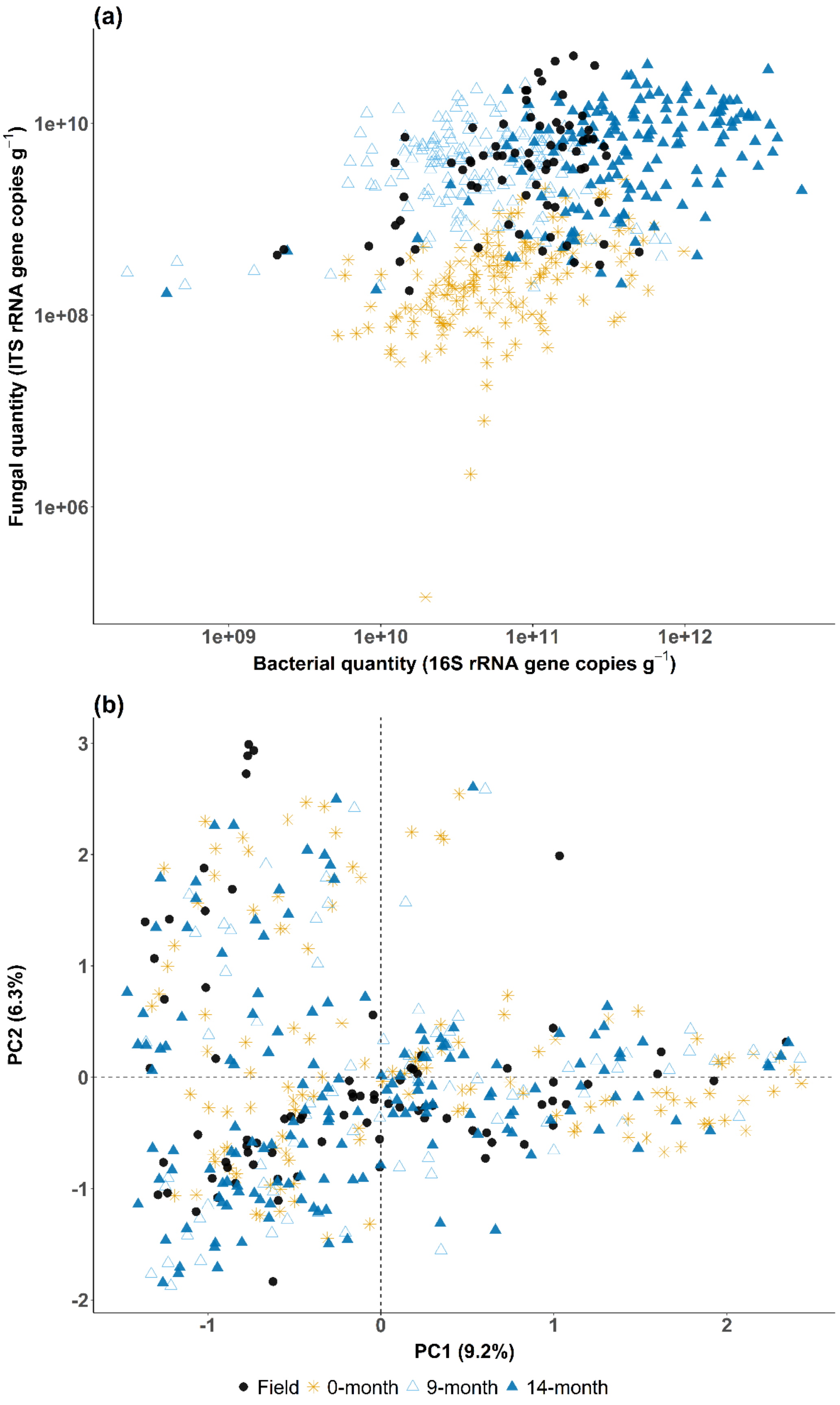
(a)
Fungal quantity (ITS rRNA gene copies g⁻¹)
1e+10
1e+08
1e+06
1e+09
1e+10
1e+11
1e+12
Bacterial quantity (16S rRNA gene copies g⁻¹)
(b)
PC2 (6.3%)
3
2
1
0
-1
-2
-1
0
1
2
PC1 (9.2%)
Field
0-month
9-month
14-month

302

Supplementary Fig. 5 Fungal and bacterial quantities and principal coordinate analysis of fungal community composition. (a) Fungal quantity (log10 scale on both y-axis and x-axis) increased from 1.1×10^5 – 2.6×10^9 (median 2.5×10^8) gene copies g^{-1} at the beginning of the incubation (0-month), to 1.9×10^8 – 2.6×10^{10} (median 4.0×10^9) gene copies g^{-1} after nine months of incubation (9-month), to 1.7×10^8 – 4.1×10^{10} (median 6.7×10^9) gene copies g^{-1} after 14 months of incubation (14-month); bacterial quantity (log10 scale on both y-axis and x-axis) increased from 5.2×10^9 – 9.8×10^{11} (median 5.8×10^{10}) gene copies g^{-1} at 0-month, and 2.1×10^8 – 7.4×10^{11} (median 3.9×10^{10}) gene copies g^{-1} at 9-month, to 3.9×10^8 –5.9×10^{12} (median 4.1×10^{11}) gene copies g^{-1} at 14-month; (b) Principal coordinate analysis based on Hellinger distance of species abundance demonstrating similarities in overall fungal community composition between the field incubation and lab incubation and over different time periods in the lab. Black dot, yellow star, blue hollow triangle, and blue solid triangle represent microbial data from the field incubation, and 0, 9, and 14 months of lab incubation, respectively. Source data are provided as a Source Data file.

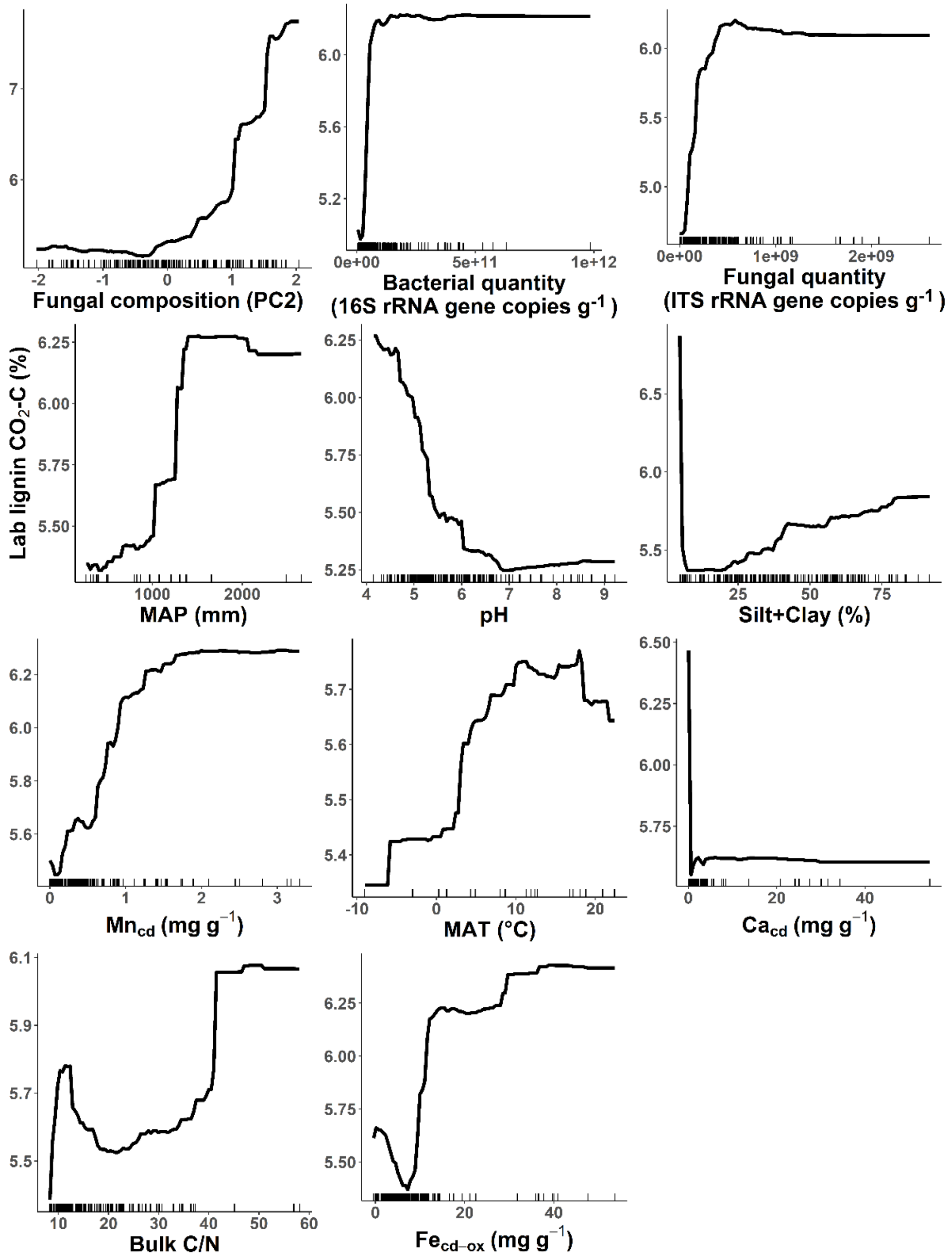

Supplementary Fig. 6 The marginal effects of predictors on the predicted lab lignin decomposition in partial dependence (PD) plots of the random forest model (RFM). Plots are ordered in a descending order of %IncMSE values (Fig. 4). Predictors correspond to those in Supplementary Table 2. Source data are provided as a Source Data file.

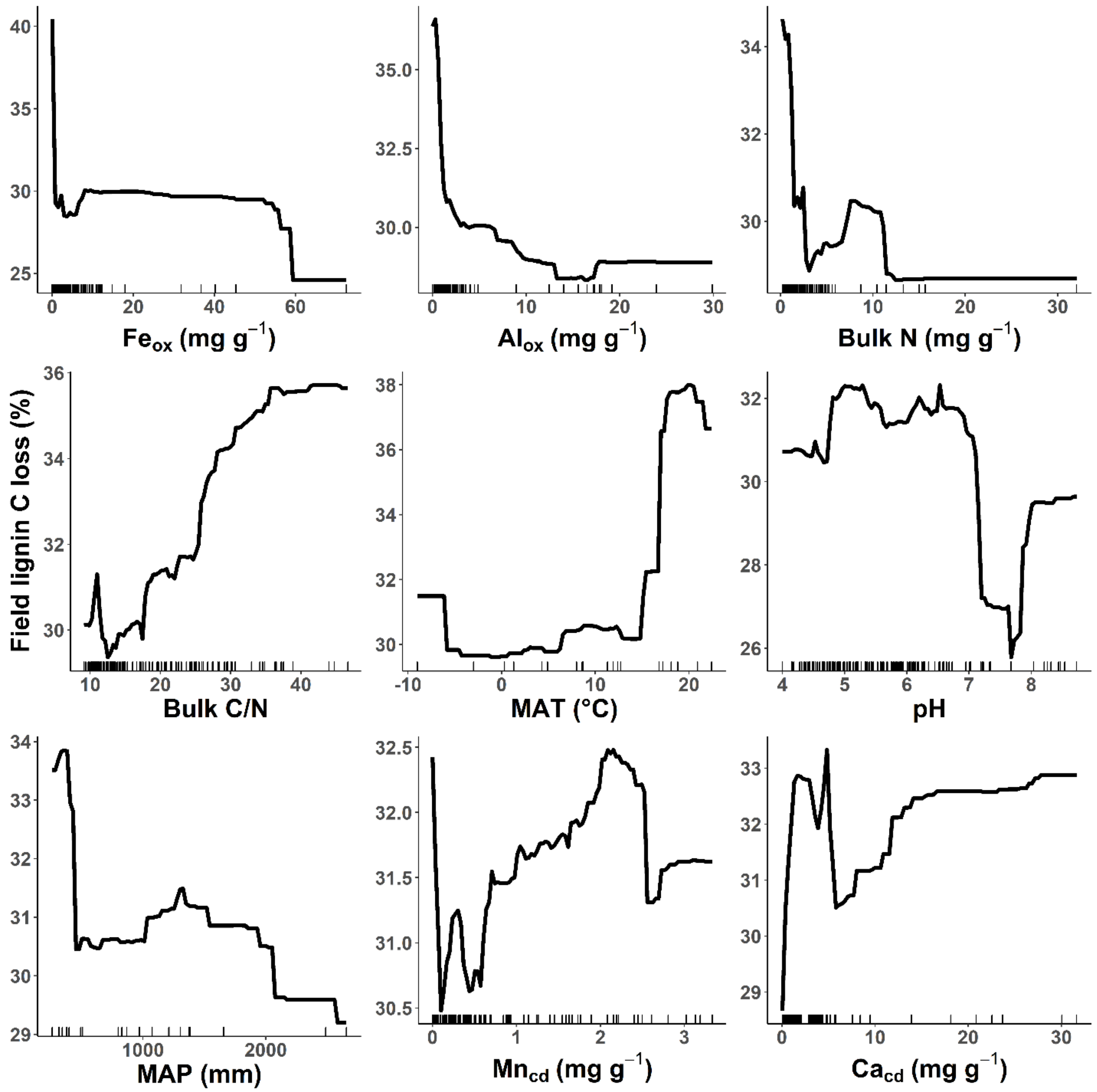

Supplementary Fig. 7 The marginal effects of predictors on the predicted field lignin decomposition in partial dependence (PD) plots of the random forest model (RFM). Plots are ordered in a descending order of %IncMSE values (Fig. 4). Predictors correspond to those in Supplementary Table 2. Source data are provided as a Source Data file.

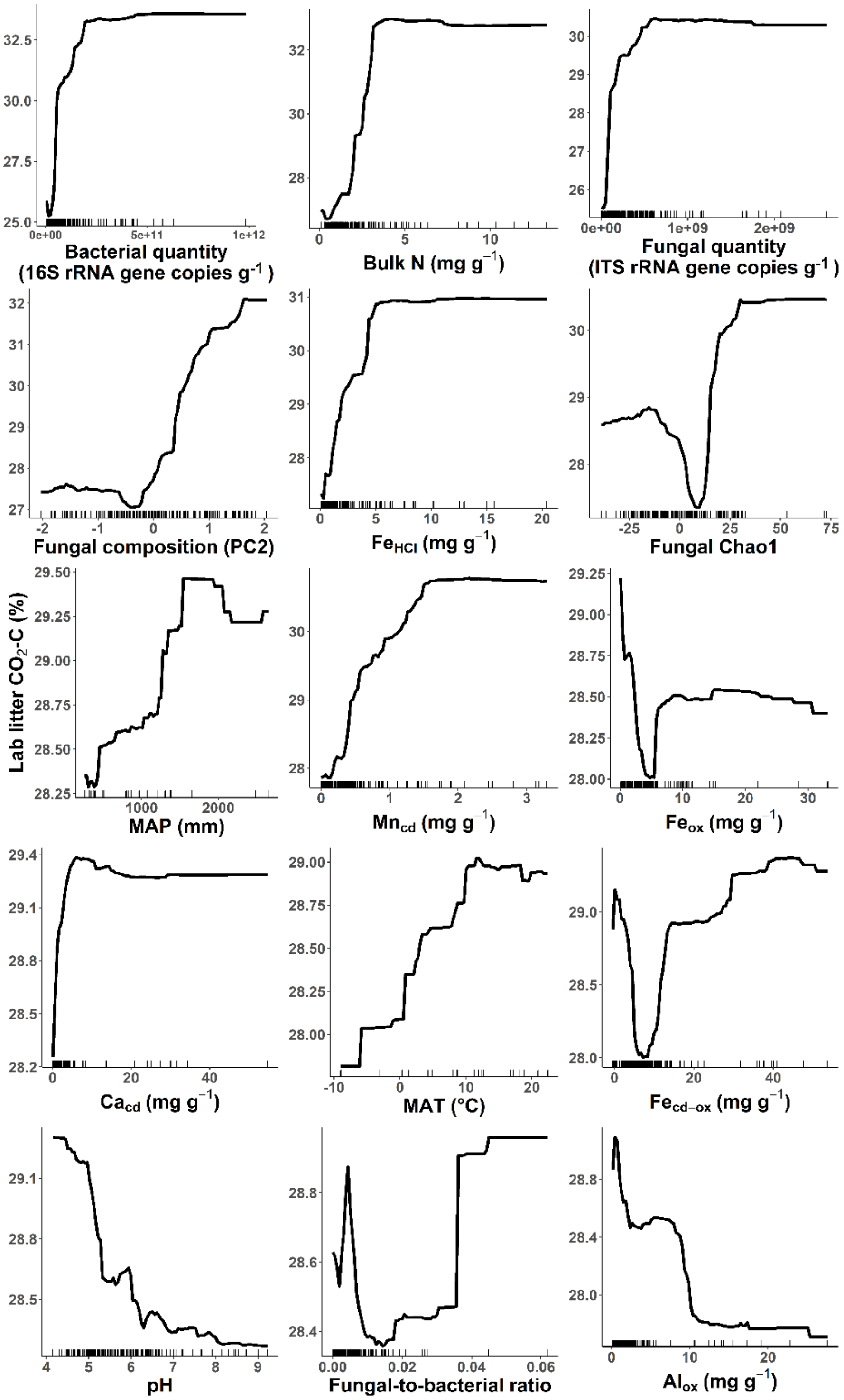

Supplementary Fig. 8 The marginal effects of predictors on the predicted litter decomposition in partial dependence (PD) plots of the random forest model (RFM). Plots

are ordered in a descending order of %IncMSE values (Fig. 4). Predictors correspond to those in Supplementary Table 2. Source data are provided as a Source Data file.

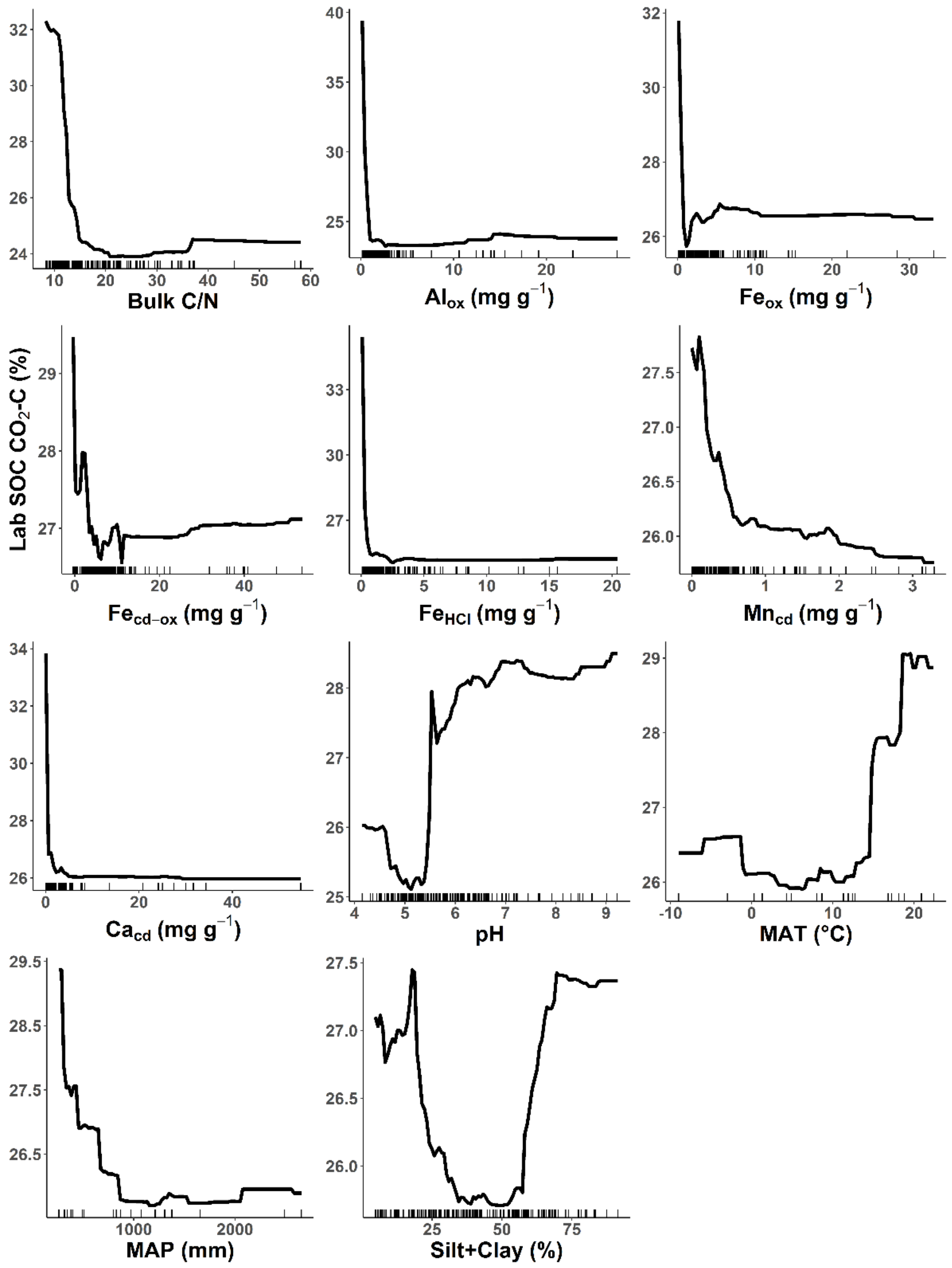

Supplementary Fig. 9 The marginal effects of predictors on the predicted SOC decomposition in partial dependence (PD) plots of the random forest model (RFM). Plots are ordered in a descending order of %IncMSE values (Fig. 4). Predictors correspond to those in Supplementary Table 2. Source data are provided as a Source Data file.

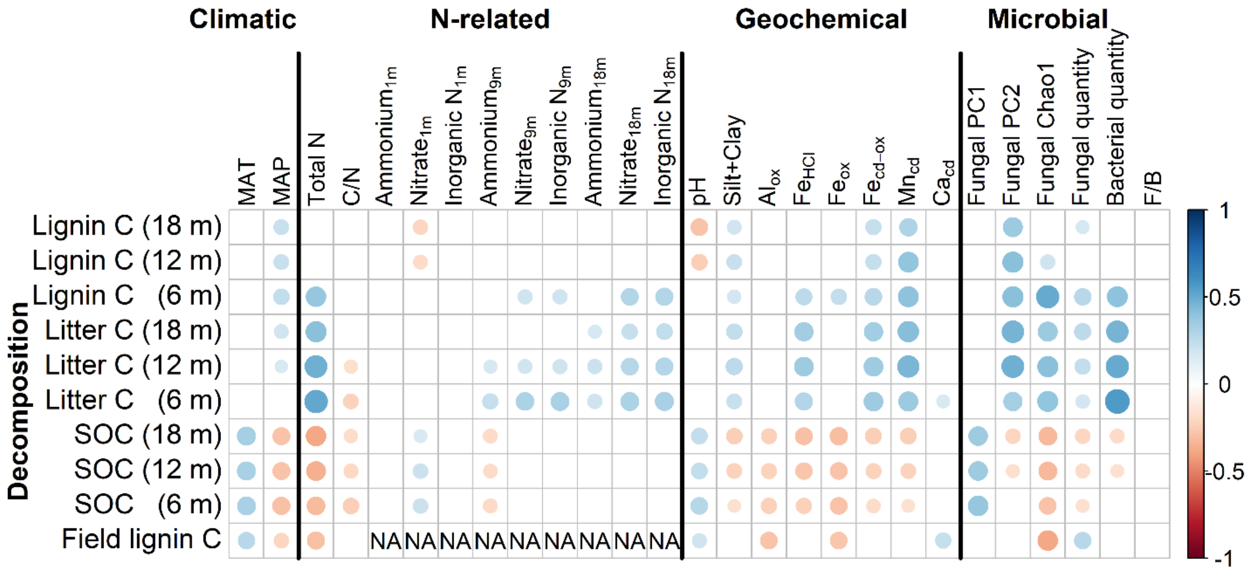

Supplementary Fig. 10 Pairwise correlations of cumulative C decomposition from lignin, litter and SOC after 18, 12, and 6 months and field lignin decomposition with climatic, N-related, geochemical and microbial predictors. Predictors correspond to those in Supplementary Table 2. Fungal composition was represented by PC1 and PC2. Significant variables calculated using a two-tailed test of significance at $P < 0.05$ using Student's t-distribution are shown in circles. Circle size and darkness indicates relationship strength; circle color indicates relationship direction (blue is positive, red is negative). The NA indicates that inorganic N data was not available for the field incubation. Source data are provided as a Source Data file.

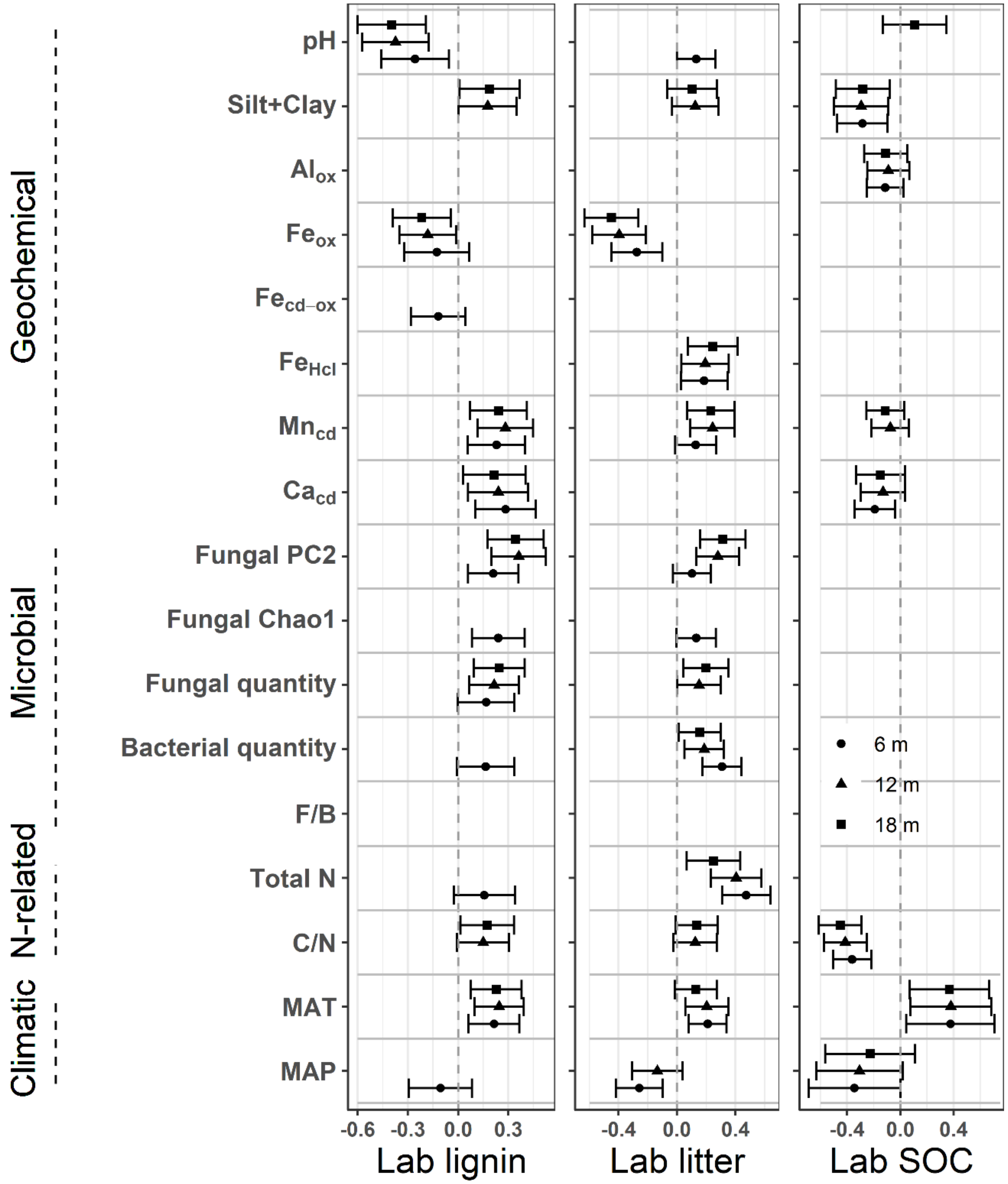

Supplementary Fig. 11 Effects of predictors on cumulative lab lignin (left), litter (middle) and SOC decomposition after 18 (square), 12 (triangle), and 6 (circle) months estimated from linear models. Linear regression models (as opposed to linear mixed models) were used for lignin and litter decomposition because the random effect of site was uninformative for the 6 and 12 month datasets whereas linear mixed models were still used for SOC decomposition.

Predictors correspond to those in Supplementary Table 2. For lignin decomposition, $R^2_{18\ m}$ = 0.44, $R^2_{12\ m}$ = 0.47, $R^2_{6\ m}$ = 0.47; for litter decomposition, $R^2_{18\ m}$ = 0.51, $R^2_{12\ m}$ = 0.58, $R^2_{6\ m}$ = 0.60; for SOC decomposition, $R^2_{18\ m\ fixed}$ = 0.43, $R^2_{18\ m\ model}$ = 0.71, $R^2_{12\ m\ fixed}$ = 0.42, $R^2_{12\ m\ model}$ = 0.72, $R^2_{6\ m\ fixed}$ = 0.41, $R^2_{6\ m\ model}$ = 0.79. Data are presented as standardized regression coefficients ± their error bars representing 95% confidence intervals (± 2 standard error). Variables were removed from the models according to AIC using stepwise backward selection. Inorganic N variables were not important for the lab incubation. For cumulative lignin decomposition, n = 146, 146, and 143 over 18, 12, and 6 months, respectively. For cumulative litter decomposition, n = 145, 144, and 145 over 18, 12, and 6 months, respectively. For cumulative SOC decomposition, n = 156, 156, and 156 over 18, 12, and 6 months, respectively. Source data are provided as a Source Data file.

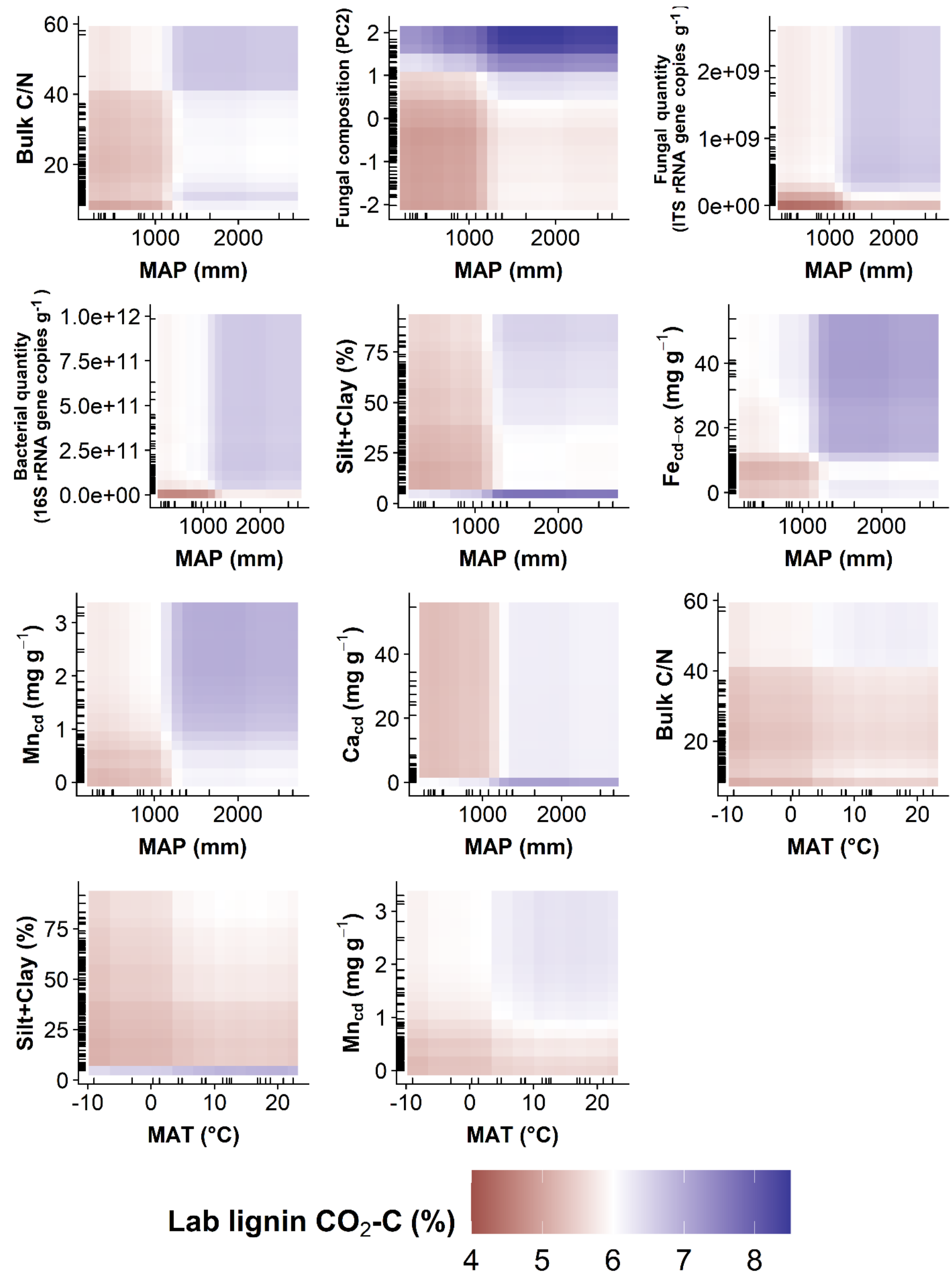
Bulk C/N
MAP (mm)
Fungal composition (PC2)
Fungal quantity (ITS rRNA gene copies g^{-1})
Bacterial quantity (16S rRNA gene copies g^{-1})
Silt+Clay (%)
Fe_{cd-ox} (mg g^{-1})
Mn_{cd} (mg g^{-1})
Ca_{cd} (mg g^{-1})
MAT (°C)
Lab lignin CO_2-C (%)

Supplementary Fig. 12 The joint effects of climatic and other predictors on the predicted lab lignin decomposition in two-way partial dependence (PD) plots in the random forest model (RFM). Interactions with H-statistic > 0.05 are presented. Lignin decomposition is back-transformed (original scale) for an easier interpretation. Color indicates strength of predicted lignin decomposition (blue is stronger, white is intermediate, red is weaker). Source data are provided as a Source Data file.

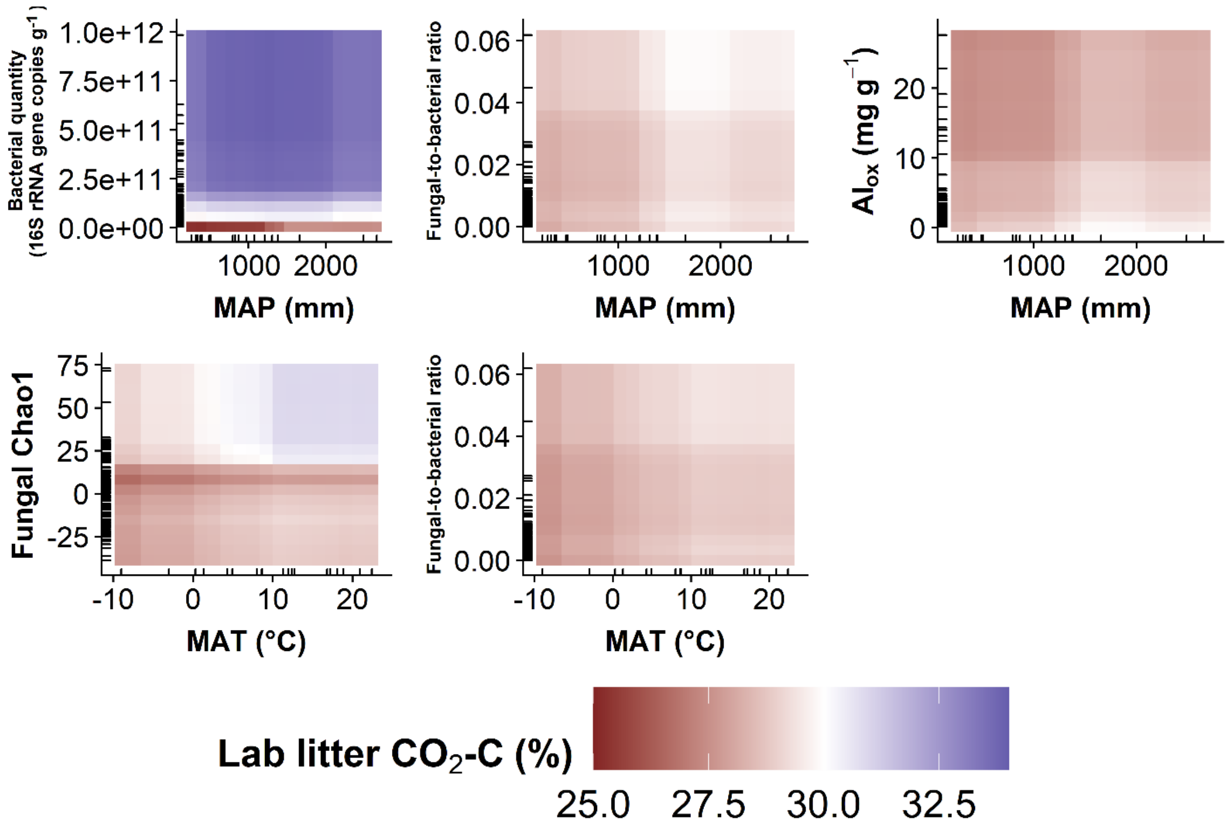

Supplementary Fig. 13 The joint effects of climatic and other predictors on the predicted lab litter decomposition in two-way partial dependence (PD) plots in the random forest model (RFM). Interactions with H-statistic > 0.05 are presented. Color indicates strength of predicted litter decomposition (blue is stronger, white is intermediate, red is weaker). Source data are provided as a Source Data file.

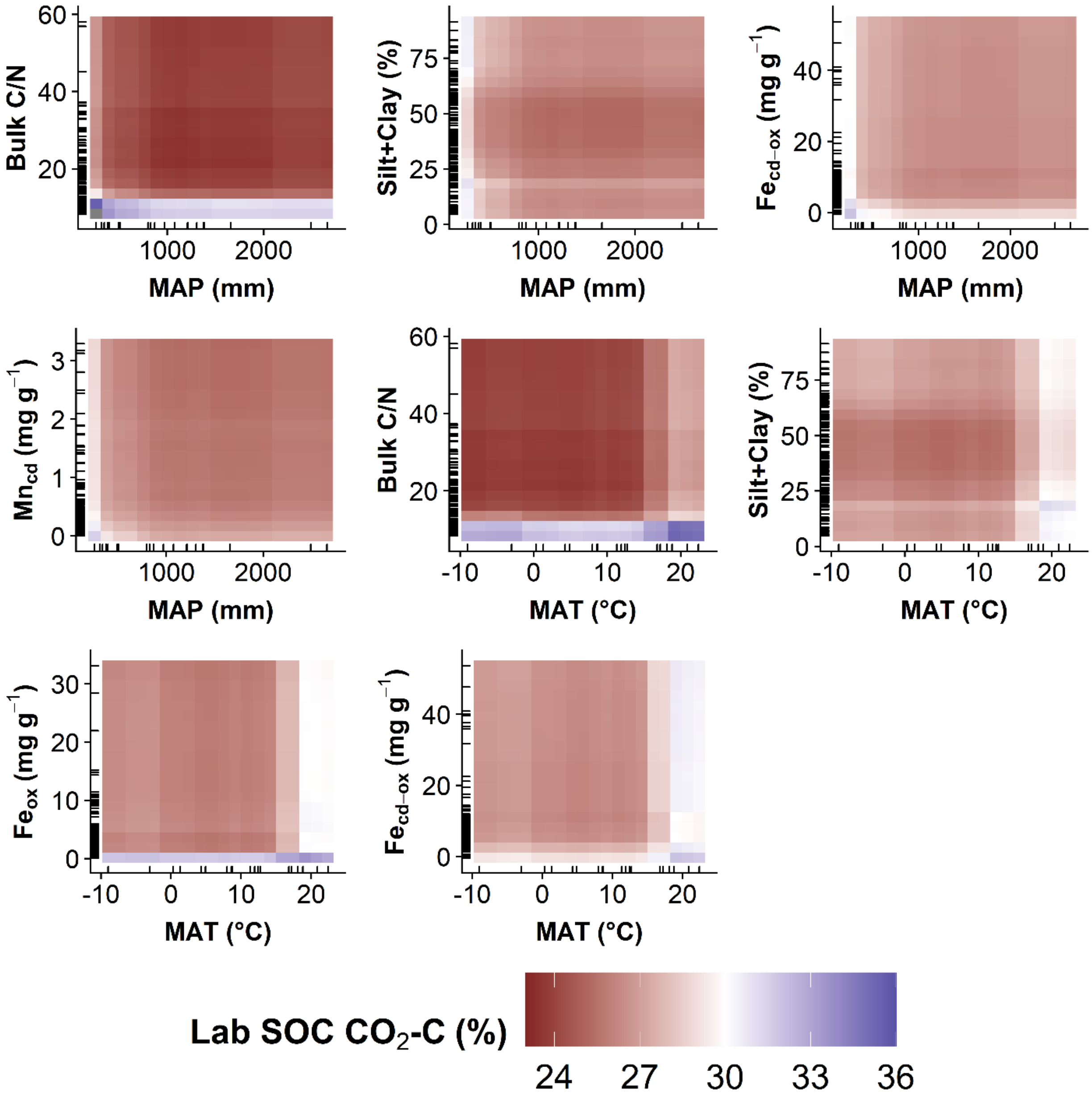

Supplementary Fig. 14 The joint effects of climatic and other predictors on the predicted lab SOC decomposition in two-way partial dependence (PD) plots in the random forest model (RFM). Interactions with H-statistic > 0.05 are presented. Color indicates strength of predicted SOC decomposition (blue is stronger, white is intermediate, red is weaker). Source data are provided as a Source Data file.

Supplementary References

1. Kirk, T. K. & Brunow, G. Synthetic ^{14}C-labeled lignins. in *Methods in Enzymology* vol. 161 65–73 (Academic Press, MA, 1988).
2. Huang, W., Ye, C., Hockaday, W. C. & Hall, S. J. Trade-offs in soil carbon protection mechanisms under aerobic and anaerobic conditions. *Glob. Chang. Biol.* **26**, 3726–3737 (2020).
3. Hall, S. J., Silver, W. L., Timokhin, V. I. & Hammel, K. E. Lignin decomposition is sustained under fluctuating redox conditions in humid tropical forest soils. *Glob. Chang. Biol.* **21**, 2818–2828 (2015).
4. Huang, W. *et al.* Enrichment of lignin-derived carbon in mineral-associated soil organic matter. *Environ. Sci. Technol.* **53**, 7522–7531 (2019).
5. Hall, S. J., Huang, W. & Hammel, K. E. An optical method for carbon dioxide isotopes and mole fractions in small gas samples: Tracing microbial respiration from soil, litter, and lignin. *Rapid Commun. Mass Spectrom*. **31**, 1938–1946 (2017).
6. Bowling, D. R., Pataki, D. E. & Randerson, J. T. Carbon isotopes in terrestrial ecosystem pools and CO_2 fluxes. *New Phytol.* **178**, 24–40 (2008).
7. Breiman, L. Random forests. *Mach. Learn.* **45**, 5–32 (2001).
8. Kursa, M. B. & Rudnicki, W. R. Feature selection with the Boruta package. *J. Stat. Softw.* **36**, 1–13 (2010).
9. Evans, J. S. & Murphy, M. A. *rfUtilities*. R package version 2.1-5. (2019).
10. Liaw, A. & Wiener, M. Classification and regression by random forest. *R News* **2/3**, 18–22 (2002).

11. Friedman, J. H. & Popescu, B. E. Predictive learning via rule ensembles. *Ann. Appl. Stat.* **2**, 916–954 (2008).

12. Wood, S. N. *Generalized Additive Models: An Introduction with R, Second Edition*. (Chapman and Hall/CRC, NY, 2017).

13. Nguyen, N. H. *et al.* FUNGuild: An open annotation tool for parsing fungal community datasets by ecological guild. *Fungal Ecol.* **20**, 241–248 (2016).

14. SanClements, M. *et al.* Collaborating with NEON. *BioScience* **70**, 107–107 (2020).
